# Molecular surveillance of influenza A virus in Saudi Arabia: whole-genome sequencing and metagenomic approaches

**DOI:** 10.1128/spectrum.00665-24

**Published:** 2024-06-21

**Authors:** Iman Dandachi, Abdulrahman Alrezaihi, Dashty Amin, Nurah AlRagi, Bader Alhatlani, Abdulwahab Binjomah, Kholoud Aleisa, Xiaofeng Dong, Julian A. Hiscox, Waleed Aljabr

**Affiliations:** 1 Research Center, King Fahad Medical City, Riyadh, Saudi Arabia; 2 Institute of Infection, Veterinary and Ecological Sciences, University of Liverpool, Liverpool, United Kingdom; 3 Faculty of Health Sciences, Qaiwan International University, Sulaymaniyah, Kurdistan Region, Iraq; 4 Pathology and Clinical Laboratory Medicine, King Fahad Medical City, Riyadh, Saudi Arabia; 5 Unit of Scientific Research, Applied College, Qassim University, Buraydah, Saudi Arabia; 6 Riyadh Regional Laboratory, Riyadh Ministry of Health, Riyadh, Saudi Arabia; Children's National Hospital, George Washington University, Washington, DC, USA

**Keywords:** metatranscriptomics, nanopore sequencing, influenza A, clade, nasopharyngeal microbiota

## Abstract

Outbreaks of influenza A viruses are generally seasonal and cause annual epidemics worldwide. Due to their frequent reassortment and evolution, annual surveillance is of paramount importance to guide vaccine strategies. The aim of this study was to explore the molecular epidemiology of influenza A virus and nasopharyngeal microbiota composition in infected patients in Saudi Arabia. A total of 103 nasopharyngeal samples from 2015 and 12 samples from 2022 were collected from patients positive for influenza A. Sequencing of influenza A as well as metatranscriptomic analysis of the nasopharyngeal microbiota was conducted using Oxford Nanopore sequencing. Phylogenetic analysis of hemagglutinin, neuraminidase segments, and concatenated influenza A genomes was performed using MEGA7. Whole-genome sequencing analysis revealed changing clades of influenza A virus: from 6B.1 in 2015 to 5a.2a in 2022. One sample containing the antiviral resistance-mediating mutation S247N toward oseltamivir and zanamivir was found. Phylogenetic analysis showed the clustering of influenza A strains with the corresponding vaccine strains in each period, thus suggesting vaccine effectiveness. Principal component analysis and alpha diversity revealed the absence of a relationship between hospital admission status, age, or gender of infected patients and the nasopharyngeal microbial composition, except for the infecting clade 5a.2a. The opportunistic pathogens *Staphylococcus aureus*, *Streptococcus pneumoniae*, *Haemophilus influenzae,* and *Moraxella catarrhalis* were the most common species detected. The molecular epidemiology appears to be changing in Saudi Arabia after the COVID-19 pandemic. Antiviral resistance should be carefully monitored in future studies. In addition, the disease severity of patients as well as the composition of the nasopharyngeal microbiota in patients infected with different clades should also be assessed.

**IMPORTANCE:**

In this work, we have found that the clade of influenza A virus circulating in Riyadh, KSA, has changed over the last few years from 6B.1 to 5a.2a. Influenza strains clustered with the corresponding vaccine strains in our population, thus emphasizing vaccine effectiveness. Metatranscriptomic analysis showed no correlation between the nasopharyngeal microbiome and the clinical and/or demographic characteristics of infected patients. This is except for the 5a.2a strains isolated post-COVID-19 pandemic. The influenza virus is among the continuously evolving viruses that can cause severe respiratory infections. Continuous surveillance of its molecular diversity and the monitoring of anti-viral-resistant strains are thus of vital importance. Furthermore, exploring potential microbial markers and/or dysbiosis of the nasopharyngeal microbiota during infection could assist in the better management of patients in severe cases.

## INTRODUCTION

Influenza viruses have been and are still a major source of morbidity and mortality worldwide, affecting large segments of the human population annually (1). Influenza infection often presents as a mild respiratory illness involving the upper respiratory tract and is characterized by the sudden onset of fever, runny nose, sore throat, cough, headache, fatigue, and muscle pain. However, in many cases, influenza is manifested as a severe or lethal pneumonia either owing to the virus itself or due to a secondary bacterial infection of the lower respiratory tract (2).

Influenza viruses belong to the Orthomyxoviridae family and are characterized by an enveloped virus particle containing a segmented genome consisting of negative-sense, single-stranded RNA (3). In this family, viruses are categorized into four types: influenza A, B, C, and D. Influenza A is the most common type known to circulate and cause seasonal epidemics in humans, in addition to type B (4). Based on the antigenicity of the two surface proteins, hemagglutinin (HA) and neuraminidase (NA), influenza A viruses are further classified into subtypes. There are currently 18 and 11 different subtypes of hemagglutinin and neuraminidase, respectively (4). While environmental factors play a considerable role in influencing the seasonality of influenza outbreaks (5), other non-environmental factors can also affect seasonal epidemics (6). Indeed, the influenza A virus has a high mutation rate, which allows it to evolve rapidly and overcome host barriers (7). While all eight segments evolve continuously, the HA and NA glycoprotein segments evolve more rapidly. This evolution is powered by antigenic shift and antigenic drift (8). Such evolution can influence the virus-host specificity/pathogenicity, affecting epidemiological behavior and may result in new human pandemics (9). For this purpose, yearly influenza virus surveillance is important to provide essential data for the reformulation of annual influenza vaccines (10).

Next-generation sequencing including nanopore is increasingly being used for the molecular surveillance of viruses (11). The aim of this study was to explore the genetic diversity of influenza A virus from historical samples as well as samples taken after the COVID-19 pandemic using Oxford Nanopore long read length sequencing. A second objective was to investigate the background context of the nasopharyngeal microbiota during influenza A infection in the Saudi population.

## MATERIALS AND METHODS

### Samples and data collection

A total of 103 nasopharyngeal aspirates were collected from patients positive for the influenza A virus at King Fahad Hospital Diagnostic Laboratory during 2014–2015, and 12 samples were collected in 2022 from a referral laboratory also located in Riyadh. The demographic and clinical characteristics of included patients were retrieved from their medical records. These included age, gender, nationality, admission status (inpatient or outpatient), length of hospital stay, presence of underlying comorbidities, co-infection with other viruses, symptoms, diagnosis, and treatment.

### RNA extraction and DNase treatment

RNA was extracted from 115 samples collected during the study periods. RNA extraction was conducted using the QIAamp Viral RNA Mini Kit (Qiagen). Extracted RNA was then subjected to DNase treatment using the TURBO DNA-free Kit (Invitrogen, Vilnius, Lithuania). Thereafter, the extracted RNA was quantified using the Qubit RNA High Sensitivity, Broad Range Assay (32852; Qiagen).

### cDNA synthesis and PCR amplification

cDNA was generated using Superscript IV reverse transcriptase (18090010; ThermoFisher) and the Uni12(M) primer 5′-AGCRAAAGCAGG-3′ (12). For nanopore sequencing, samples were first screened using the M and NS segments. The eight segments of influenza A were then amplified using the Q5 High-Fidelity DNA Polymerase (M0491; New England BioLabs) and eight sets of primers (Tables S1 and S2) (12).

### Sequence-independent, single-primer amplification

For the identification of viral and bacterial transcripts in nasopharyngeal (NP) samples, sequence-independent, single-primer amplification (SISPA) was conducted, as previously described (13). Qubit double-stranded DNA high-sensitivity assay (Q32851; Invitrogen) was then used to quantify amplified products.

### Minion sequencing

For sequencing the eight segments of influenza A, the protocol of the “PCR tiling of COVID-19 virus, Version:PTC_9096_v109_revD_06Feb2020” was optimized as per the influenza A virus genome and applied. Generated amplicons (from either influenza genomes or SISPA products) were then pooled and purified with AMPureXP beads (A63882; Beckman Coulter). The library was prepared as per the sequencing by ligation protocol with native barcodes for multiplexing (SQK-LSK109; Oxford Nanopore Technologies) and was then added to a flow cell (R9.4.1) connected to an MInIT device, and sequencing was initiated via MinKNOW.

### Bioinformatic analysis

Using Guppy V6.5.7, Fast5 files were basecalled. For the influenza A genomes, library reads were filtered by expected amplicon size and aligned to the NCBI influenza A virus [A/California/07/2009(H1N1)] GCF_001343785.1 using minimap2 (V2.26). Alignment files were then sorted/indexed using SAMtools (V1.13). Picard MarkDuplicates was used to remove amplification duplicates (V2.18.2). Sequencing coverage was derived from BAM Coverage Plotter. Consensus sequences of PB2, PB1, PA, HA, NA, and NP were retrieved with Raven (V1.8.0). M and NS segments were retrieved using rnaviralSPAdes (V3.15.4). Minor allele variants, i.e., amino acid changes with a frequency ≤ 0.05 for HA and NA were assessed using iVar (V1.4.2). Next clade was used to determine the clades of each influenza A virus (14).

For metatranscriptomic analysis, using Kraken2, metatranscriptomic reads were assessed (15). The abundance of genus and phyla was estimated using Bracken (V2.8). Alpha and Beta diversities were then calculated using Krakentools (V1.2). Using Rstudio and the calculated beta diversity, principal component analysis (PCA) and accordingly ggplot2 were performed. Using Krakentools, Kraken reports were converted to a MetaPhlan style report, and a heatmap was generated showing the most abundant 15 species using “generate heatmap” (V2.6.0). For antimicrobial resistance and virulence gene identification, VFDB and MEGARes databases were used using Abricate (V.1.0.1); hits with at least 80% coverage and 75% identity were considered.

### Phylogenetic analysis

Consensus sequences for each sample were concatenated in the following order: PB2, PB1, PA, HA, NP, NA, M, and NS. In addition, HA and NA segments were blasted in NCBI, and the most closely related sequences were retrieved. Reference influenza A/H1N1pdm09 vaccine strains (California/07/2009 and Michigan/45/2015) and the new reference vaccine strains were retrieved from the NCBI Influenza Resource Database and the Global Initiative on Sharing All Influenza Data, respectively (16, 17). Using MEGA7, nucleotide sequences were aligned with ClustalW, and a phylogenetic tree was constructed according to the best-fit nucleotide substitution model “Tamura Nei model” (18, 19).

## RESULTS

### General characteristics of the studied population

Out of 115 samples, 44 showed successful amplification of the M and NS segments. These included 32 samples collected during 2014–2015 and 12 collected in 2022. Table S3; Table 1 show the demographic and clinical characteristics of included subjects, respectively. The majority were females and <40 years. Most of the subjects were outpatients (*n* = 18), with only 13 being hospitalized. Viral co-infection was detected in three patients, with the co-detected viruses being rhinovirus, adenovirus, and respiratory syncytial virus (RSV) (Table 1). For the 2022 samples, no metadata were available except for the gender, age, and nationality of 11 patients.

**TABLE 1:** Clinical and microbiota characteristics of the studied population^*a*^

Sample	Admission status	LOS (days)	Comorbidities	Symptoms	Viral co-infection/post-COVID-19	Diagnosis	Treatment	Viruses detected by Meta Seq	Most common Phyla detected across all samples	Most common Genera detected across all samples	Most common Species detected across all samples	Resistance/ virulence genes
**2**	Outpatient			Fever	No		Tamiflu	Influenza A	Spirochaetes	*Veilonella*	*H. influenzae*	
**5**	Inpatient	2	Recent gastritis	Fever, cough, and SOB for 3 days	No		Tamiflu and amoxicillin	Influenza A	Bacteroidetes	*Fusobacterium*	*Veilonella atypica*	
**6**	Inpatient				No			Influenza A	Fusobacteria	*Prevotella*	*S. aureus*	
**7**	HRW	8	HTN, CHF, CKD, pulmonary HTN, atrial fibrillation, hypothyroidism	Fever, mild SOB, and productive cough	No	Viral pneumonia	CFO IV, doxycycline IV, Tamiflu, and Tazocin IV	Influenza A	Chloroflexi	*Neisseria*	*M. catarrhalis*	
**9**	Inpatient	5	Pregnant at 36 weeks, anemia	Fever, vomiting, and dry cough	No		Tamiflu	Influenza A	Tenericutes	*Bacillus*	*S. pneumoniae*	
**10**	Inpatient	3	Stage IV myxoid liposarcoma	Respiratory tract infection and neutropenia	No		Tamiflu	Influenza A, HKU1	Firmicutes	*Streptococcus*	*Veillonella parvula*	
**11**	Outpatient			Sore throat, dry cough, runny nose, eye redness, and headache	No	Viral tonsilitis and bacterial conjunctivitis	Tamiflu	Influenza A	Proteobacteria	*Staphylococcus*	*H. parainfluenzae*	
**12**	Inpatient	7	Sarcoma, febrile neutropenia, thrombocytopenia, and hypotensive		RH		Tamiflu, Tazocin, and VAN	Influenza A	Actinobacteria	*Moraxella*	*Mycobacterium branderi*	RLMH, cap8E, and clpP
**13**	Inpatient	6	Pregnant	Fever with URTI	Adeno and RH			Influenza A, HKU1	Cyanobacteria	*Mycobacterium*	*Vibrio anguillarum*	
**26**	Outpatient			Fever	No		Tamiflu, paracetamol, and AZT	Influenza A		*Corynebacterium*	*Acinetobacter baumannii*	
**27**	Outpatient			Cough with fever	No		Tamiflu	Influenza A		*Vibrio*	*Pseudomonas aeruginosa*	
**31**	Outpatient			Fever with cough	No	Viral pneumonia	Tamiflu and MOX	Influenza A		*Pseudomonas*	*Escherichia coli*	
**33**	Outpatient		Bronchial asthma, pre-diabetic, and HTN	Cough and flu-like symptoms	No		Tamiflu	Influenza A		*Acinetobacter*	*Klebsiella pneumoniae*	
**34**	Inpatient	1	Pregnant	Fever	No	Viral pneumonia		Influenza A		*Escherichia*	*Salmonella enterica*	
**36**	Inpatient	8		Fever	RSV	Viral pneumonia	Tamiflu and Augmentin	Influenza A		*Haemophilus*		
**39**	Outpatient			Dry cough	No		Tamiflu	Influenza A, RSV				
**41**	Outpatient			Cough	No			Influenza A				
**49**	Outpatient			Fever	No		Tamiflu and paracetamol	Influenza A				
**53**	HRW	5	DM, HTN, and dyslipidemia	SOB, fever, and productive cough	No	Viral pneumonia, partially treated bacterial pneumonia	FEP, MOX, Ventolin, Symbicort, and Tamiflu	Influenza A				
**58**	Outpatient				No	Influenza		Influenza A, RSV				
**64**	Outpatient			Fever	No	Acute URTI with multiple sites	Tamiflu and paracetamol	Influenza A, RH				
**76**	Inpatient	7		Fever	No	Viral pneumonia		Influenza A				
**78**	Outpatient		Medically free	Fever with chills	No	Pneumonia	Moxifloxacin	Influenza A				
**81**	Outpatient		DM and metastatic adenocarcinoma	Fever	No		Tamiflu	Influenza A				
**83**	Outpatient			Fever	No	Acute URTI with multiple sites	Tamiflu and paracetamol	Influenza A				
**89**	HRW	11		Cough, SOB, orthopnea, PND, and fever	No	Community-acquired pneumonia	Tamiflu	Influenza A				
**95**	Outpatient			Cough	No		Tamiflu	Influenza A				
**98**	GPW and PICU	14	SCA with acute chest syndrome	Fever, SOB, and cough for 5 days	No	H1N1 pneumonia	CFO, AZT, VAN, MEP, and acyclovir	Influenza A, RSV, RH				
**99**	Outpatient			Fever	No	Acute bronchitis	Tamiflu, paracetamol, and MOX	Influenza A				
**102**	Outpatient			Fever	No			Influenza A, RSV				
**103**	Outpatient			Fever	No			Influenza A, RSV				
**74**					No			Influenza A				
**N1**					No			Influenza A				
**N2**					No			Influenza A				
**N8**				Fever and cough	Yes			Influenza A				
**N9**				Fever and cough	No			Influenza A				
**N10**				Fever and cough	No			Influenza A				
**N11**				Fever and cough	No			Influenza A				
**N13**				SOB and cough	No			Influenza A				
**N14**				SOB and cough	Yes			Influenza A				
**N15**					No			Influenza A				
**N17**				Fever, headache, and cough	No			Influenza A				
**N18**				Fever	No			Influenza A				
**N19**					No			Influenza A				

^*a*^ HTN = hypertension, DM = diabetes mellitus , CHF = congestive heart failure, CKD = chronic kidney disease, SOB = shortness of breath, SCA = sickle cell anemia, HRW = High-risk ward, GPW = general pediatric ward, INF = infection, ESRD = end stage renal disease, LOS = length of hospital stay, CFO = ceftriaxone, FEP = cefepime, PND = paroxysmal nocturnal dyspnea, RSV = respiratory syncytial virus, RH = rhinovirus, adeno = adenovirus, PICU = pediatric intensive care unit, URTI = upper respiratory tract infection, AZT = azithromycin, MEP = meropenem, VAN = vancomycin, MOX = Moxifloxacin, Meta seq = metatranscriptomic sequencing, N = new samples collected in 2022, Blank = data not available.

### Whole genome analysis of influenza A virus

**Fig 1:**
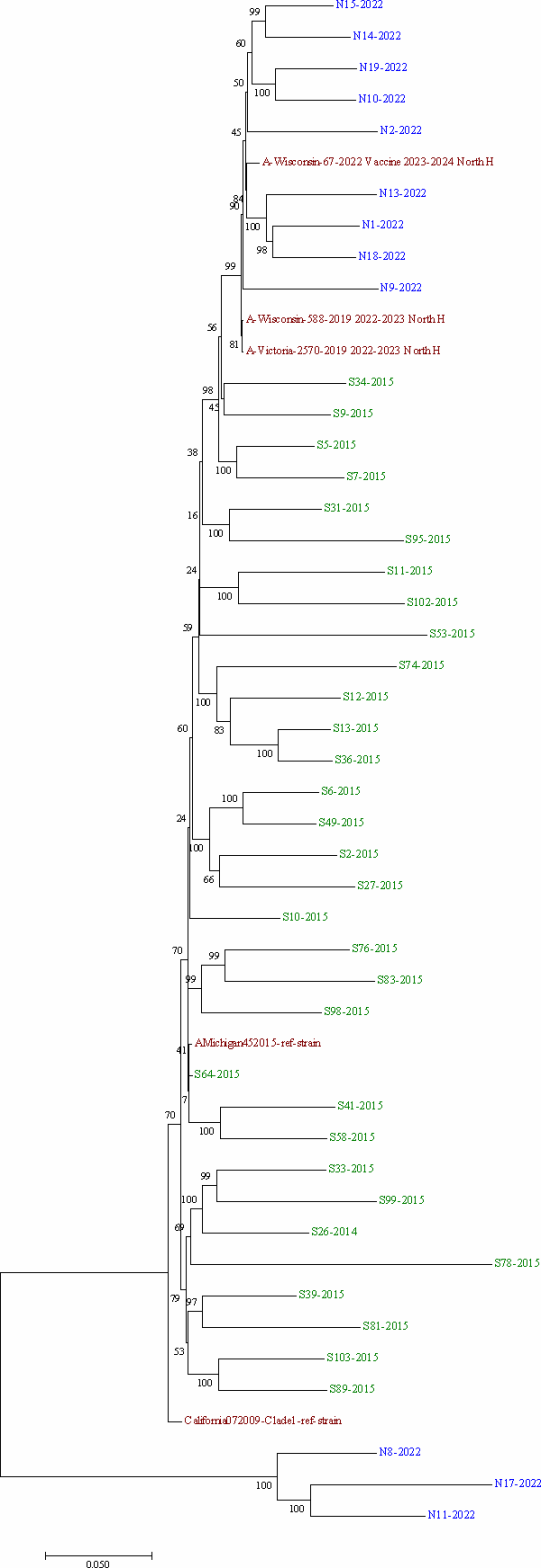
Phylogenetic tree of influenza A concatenated genomes. The evolutionary history was inferred using the maximum likelihood method based on the Tamura-Nei model. The percentage of trees in which the associated taxa clustered together is shown next to the branches. The tree is drawn to scale, with branch lengths measured in the number of substitutions per site.

The eight segments of influenza A in the 44 samples were successfully sequenced using nanopore. The sequencing coverage was homogeneous throughout all positions for HA, NP, NA, and NS segments but heterogeneous for PB2, PB1, and PA (Fig. S1). Phylogenetic analysis of concatenated genomes showed that influenza A strains from the 2014–2015 phase clustered together, along with the two reference vaccine strains Michigan2015 and California2009. On the other hand, those from 2022 clustered with the new vaccine reference strains. This is except for three from 2022 that clustered together (Fig. 1). Of the 32 strains from the 2014–2015 period, 28 strains belonged to the 6B.1 clade except for four that were 6B.2. Samples from 2022 were all from the 5a.2a clade, except for three that belonged to the 2b (N8) and 2a.3b (N17 and N11) clades.

### Phylogenetic analysis of HA, NA, and minor allele variants

**Fig 2:**
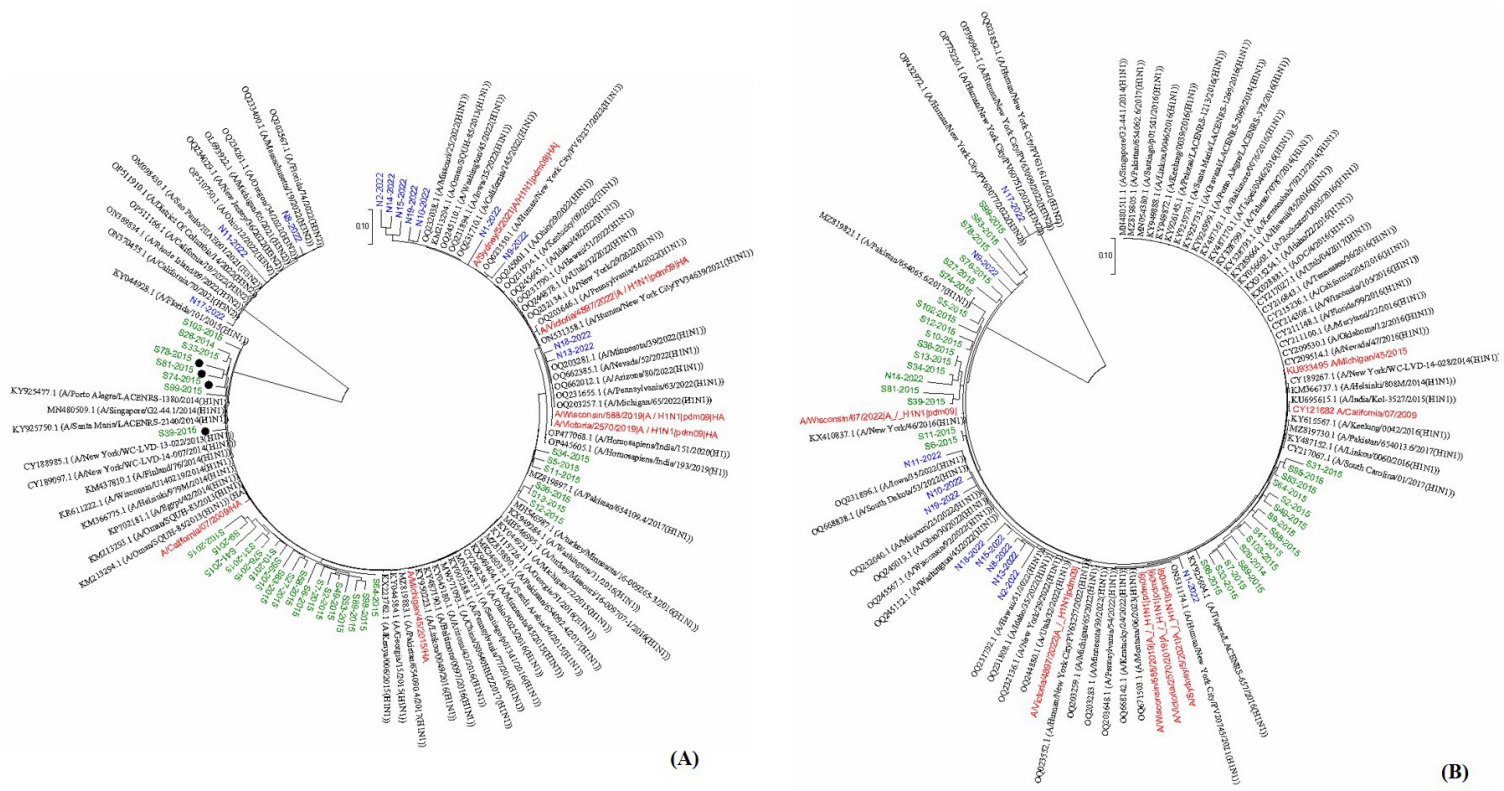
Phylogenetic tree of influenza A HA (**A**) and NA (**B**) segments. The tree is drawn to scale, with branch lengths measured in the number of substitutions per site. The evolutionary history was inferred using the maximum likelihood method based on the Tamura-Nei model.

**Fig 3:**
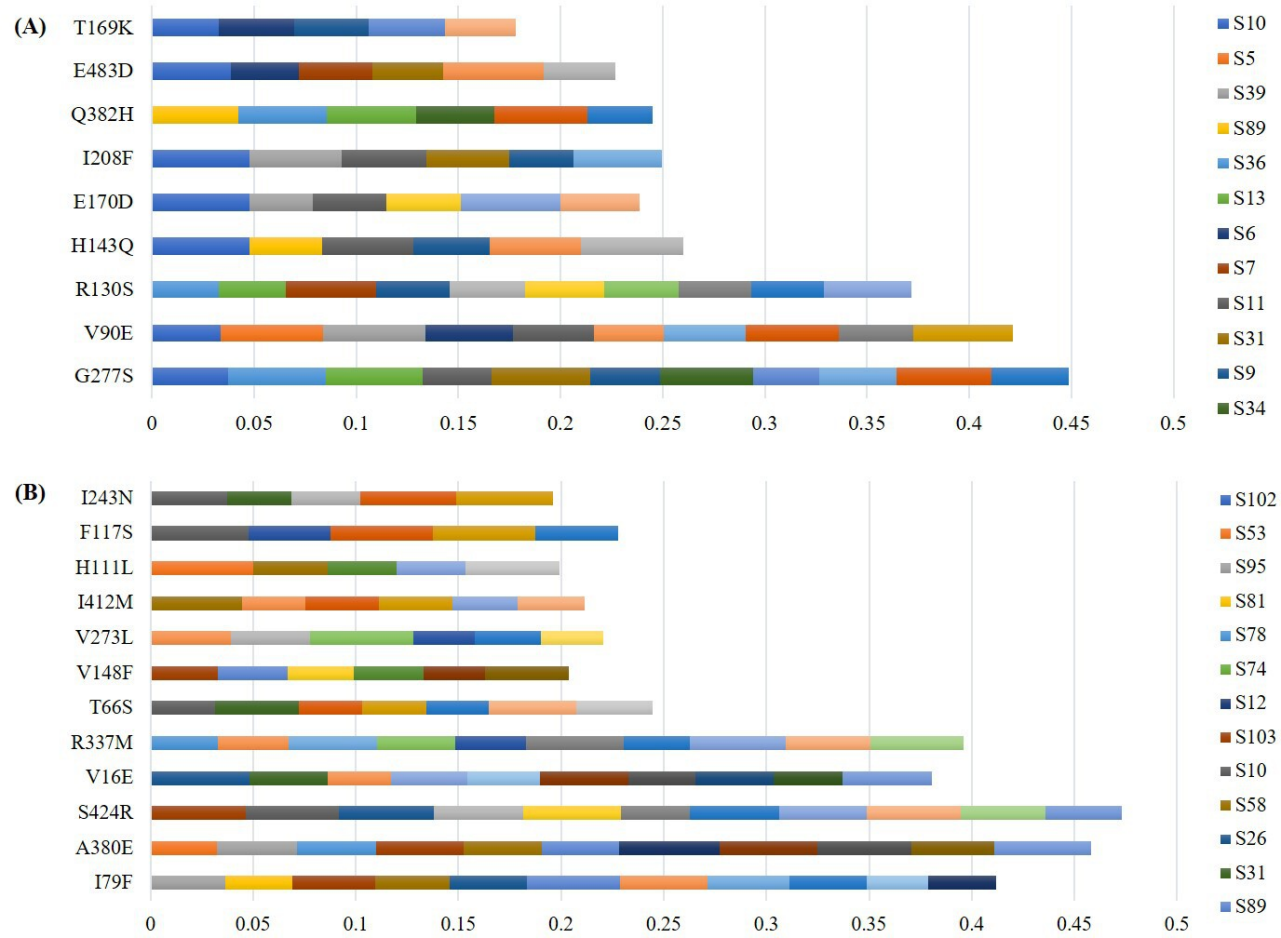
Minor allele amino acid variants detected in influenza A samples. The *X* axis represents frequency minor variants. (**A**) Variations in the HA segment and (**B**) variations in the NA segment.

Phylogenetic analysis of the HA segments from 2014 to 2015 showed that these clustered together and were closely related to H1N1 worldwide strains isolated during the same year, including ones from the Middle East. Similarly, 2022 samples were closely related to H1N1 strains isolated during the same year. This is except for three (N8, N11, and N17), which were related to H3N2 (Fig. 2A). For NA, 2022 samples were clustering with the corresponding vaccine strains. NA segment from sample N17 was closely related to H3N2. On the other hand, NA segments from 2014 to 2015 formed two clusters, with the most commonly related one being the reference vaccine strain California2009 (Fig. 2B). Minor allele variant analysis of HA showed that G277S, V90E, R130S, and H143Q were the most common variations observed (Fig. 3A). On the other hand, S424R, A380E, and R337M were the most common minor alleles observed in NA (Fig. 3B). No substitutions associated with resistance to neuraminidase inhibitors were found. This is except for sample 33, where the S247N mutation was found.

### Metagenomic analysis of influenza A samples

**Fig 4:**
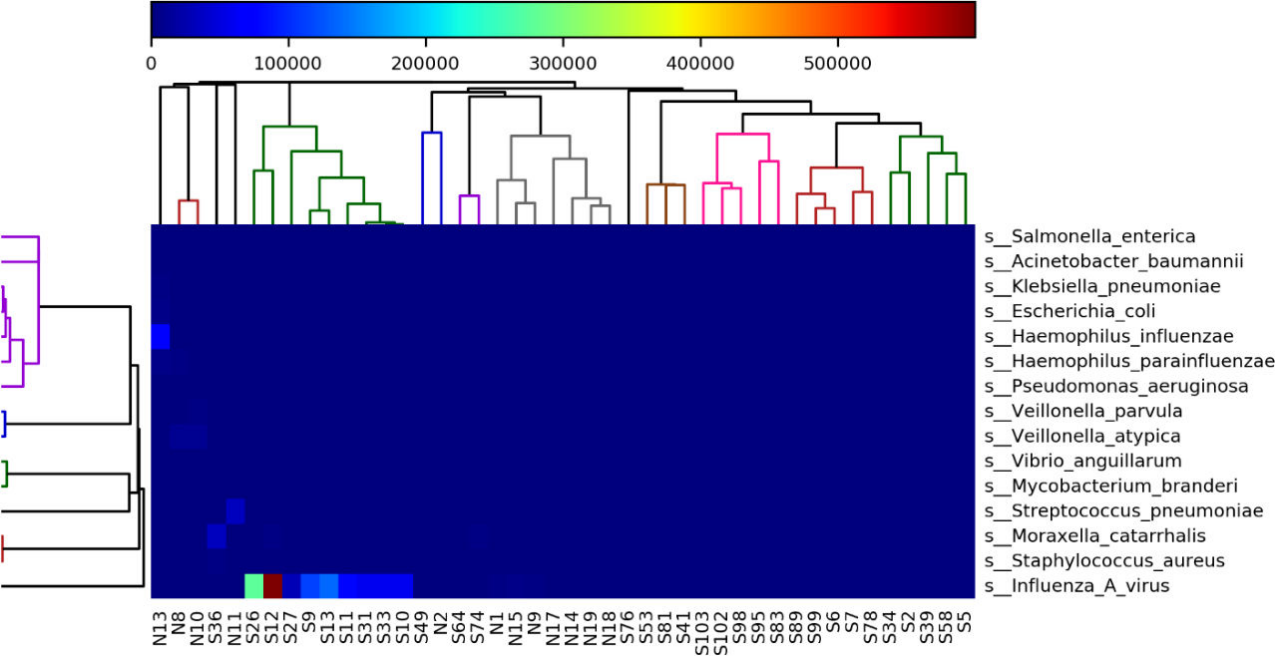
Heatmap showing the most common microbial species detected across all influenza samples in this study.

Metagenomic analysis showed that the most common bacterial species detected in the nasopharyngeal microbiota of influenza patients were *Haemophilus influenzae, Veilonella atypica, Moraxella catarrhalis*, *Streptococcus pneumoniae*, and *Staphylococcus aureus* (Fig. 4; Table 1). Influenza A was detected in all samples; other co-detected viruses belonged to the pneumoviridae family (RSV), picornaviridae (rhinovirus), and the coronaviridae (HKU1).

### Differences in microbiome composition between influenza A patients

**Fig 5:**
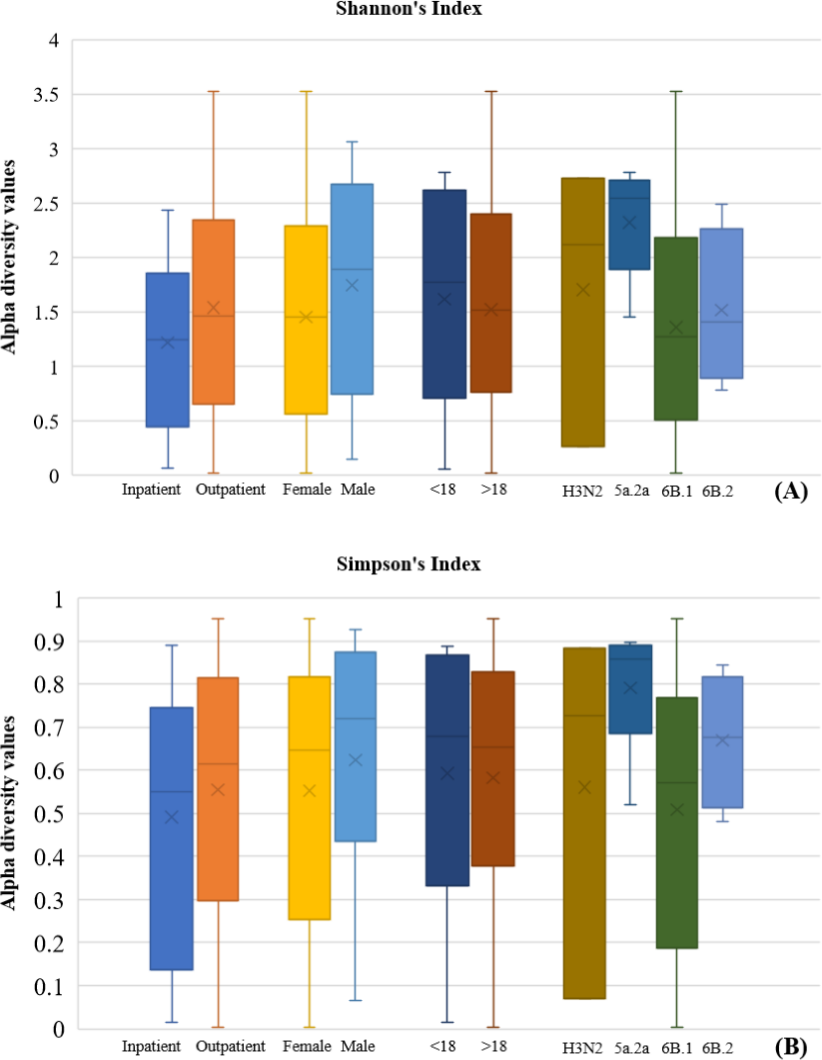
Alpha diversity of all samples at the level of hospital admission status, gender, age, and influenza A clades, based on (A) Shannon’s and (B) Simpson’s diversity indexes. Each box plot shows the maximum and minimum values and the mean “*x.*”

**Fig 6:**
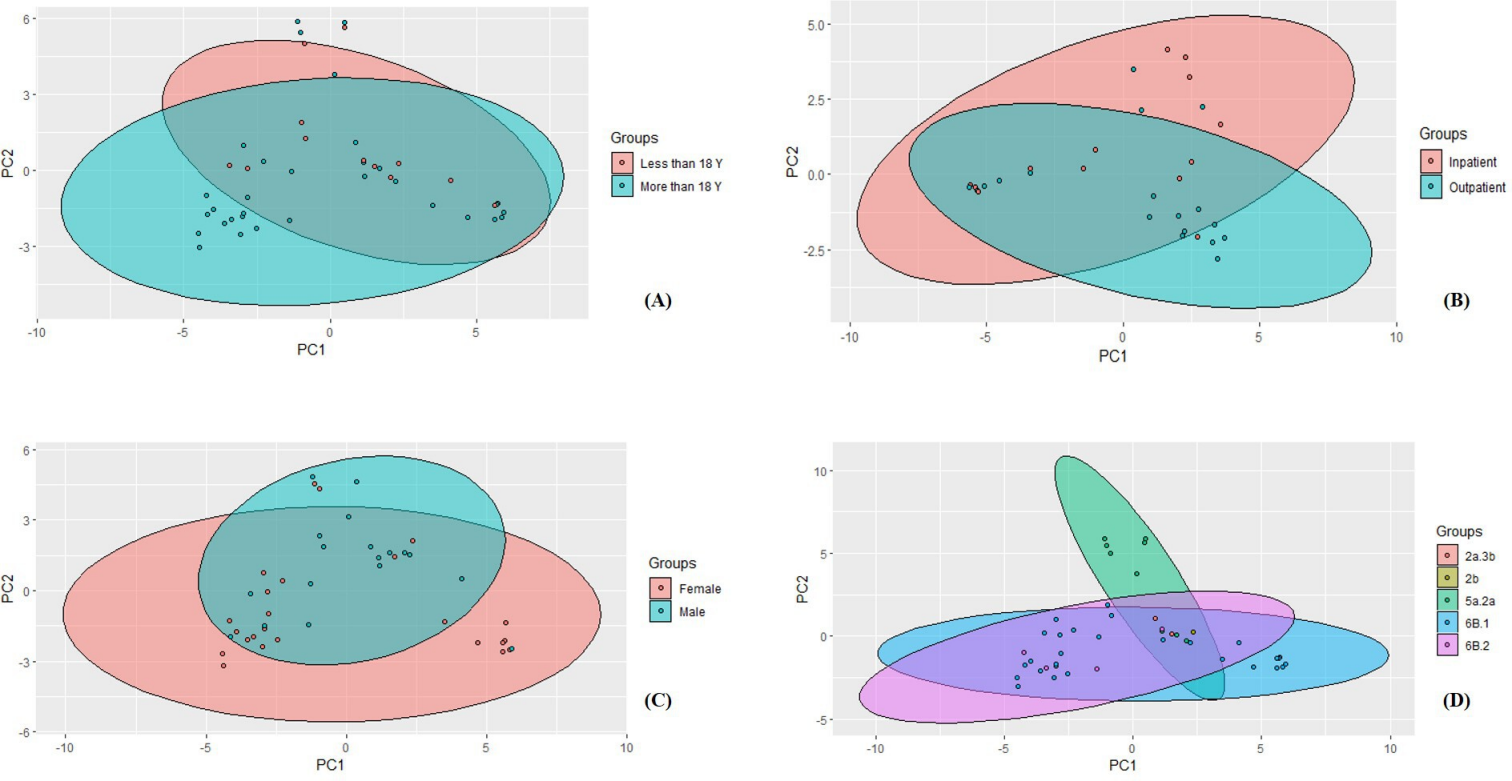
Principal component analysis of the microbial communities’ beta diversity of influenza samples that were compared based on different categorizations. (A) Age: 44 patients included, (B) hospital admission status: 32 included, (C) gender: 43 included, and (D) influenza A clades, all 44 included.

Overall, similar alpha diversity between inpatients/outpatients, females/males, and age groups at both Shannon’s and Simpsons’ indexes was observed (Fig. 5). Patients infected with clade 5a.2a had a higher mean of alpha diversity compared to other clades at both indexes. Our analysis indicated that at the genera level, *Haemophilus*, *Moraxella*, *Veilonella*, *Fusobacterium*, *Prevotella*, *Neisseria*, *Streptococcus*, and *Staphylococcus* were the most common (Table 1). At the phylum level, Proteobacteria, Firmicutes, Actinobacteria, Fusobacteria, and Bacteroidetes were the most common (Table 1). PCA indicated that influenza samples overlapped at the level of age, gender, and hospital admission and showed separate clustering only at the level of the 5a.2a clade, where 6/9 patients did not overlap with the others (Fig. 6).

### Resistome and virulome of the nasopharyngeal microbiota

Resistance and virulence genes were detected in one patient. These included 23S_rRNA_methyltransferases, aminoglycoside_O-phosphotransferase gene, and the capsular polysaccharide synthesis enzyme Cap8E and ATP-dependent Clp protease proteolytic subunit virulence genes (Table S4).

## DISCUSSION

Although occurring yearly, the influenza virus needs to be continuously monitored for the early detection of variants that can cause human pandemics as well as to guide health authorities for the proper inclusion of viral lineages in seasonal vaccines. Since the start of the COVID-19 pandemic, a global decrease in influenza virus has been noted worldwide. In the United States, for example, it has been reported that the virus circulation sharply declined within 2 weeks of the declaration of the COVID-19 emergency (20). In fact, it has been suggested that the reduction in influenza cases has resulted in reduced genetic diversity (21). In our study, we found that influenza A strains collected after COVID-19 belonged to clade 6B.1A.5a.2a, while those from 2014 to 2015 belonged to 6B.1 and 6B.2. It is worth mentioning that influenza samples collected after COVID-19 in this study were from a referral laboratory from the Riyadh region. This suggests that although these samples are few, they could give an idea about the changing epidemiology of the virus in the region. Clade 5a.2a has been reported during the same year according to the World Health Organization of the European region (22). In Saudi Arabia, no studies have yet elucidated the clades of influenza A virus, so comparisons are problematic to make. This is a general reflection of a lower amount of epidemiological monitoring of viral variants in the MEWA (Middle East West Asia) region. Our results in the pre-COVID-19 era were in accordance with other studies reported in KSA, where the H1N1 clade 6B.1 appeared to be dominant during the same period (23, 24). In fact, during 2015/2016, clade 6B.1/6B.2 became dominant worldwide and was associated with increased disease severity and hospital admissions (25). No relation between a specific influenza clade and patients’ metadata was observed in this study. The majority of our samples were from outpatients, indicating that these clades were not potentially associated with an increase in disease severity. Patients who were admitted in this study might have been hospitalized for reasons other than influenza. This is because the majority (8/14) had serious comorbidities, including atrial fibrillation, hypothyroidism, and metastatic liposarcoma. Fortunately, only one isolate in which the S247N mutation that mediates oseltamivir and zanamivir resistance was found (26). Nevertheless, the S247N mutation should be carefully monitored in future surveillance studies of influenza A in Saudi Arabia. Of all samples collected, only three strains were H3N2 and belonged to 3C.2a1b.2a.2b and 3C.2a1b.2a.2a.3b clades. Previous H3N2 detected in KSA belonged to 3c.2a1b.1 and 3c.2a1 clusters (27).

Interestingly, phylogenetic analysis revealed that the strains identified in this study clustered with the corresponding vaccine strains. Similarly, in their study, Dudin et al. (27) reported that the majority of influenza strains clustered within the same clade of the vaccine strains. Nevertheless, studies addressing influenza vaccine effectiveness in KSA are scarce. In fact, it has been estimated that when the vaccine strains are well matched antigenically with the ones in the circulating area, current influenza vaccine effectiveness is still suboptimal and ranges from 40% to 60% (28). This is in accordance with a recent study conducted in Saudi Arabia that has found that vaccine effectiveness for influenza A H1N1 and H3N2 is 39.2% and 37.4%, respectively (29). For instance, a recent study from KSA has found that H3N2 influenza A strains were the most commonly detected during 2019–2020, followed by influenza A H1N1 pdm09 (29). According to the Saudi Ministry of Health, diabetic subjects, patients with neurological disorders, chronic diseases including cardiac, renal, and liver diseases, and those who are immunodeficient or are on long-term aspirin therapy (6 months to 18 years old) are recommended and targeted for the yearly seasonal influenza vaccine uptake. This is in addition to those with morbid obesity, pregnant women, all healthcare workers, children 6 months to 5 years old, and persons who are more than 50 years old (30).

Metatranscriptomic analysis in this study revealed the detection of influenza A virus in all samples. In addition, this analysis allowed the detection of other respiratory viruses in eight samples (18.2%), which were not present in the patients’ clinical reports. The reason behind this could be either remnants of previous infection or that the routine diagnostic tests did not identify the current infections. On the other hand, we had three samples, which, according to their clinical records, were co-infected with other respiratory viruses, including adenovirus, rhinovirus, and RSV, but turned out to be negative for these latter with metatranscriptomic sequencing. One possible reason for this could be the low viral load of these viruses in the samples, complemented with the large bacterial background upon sequencing, which hindered the sequencing of the viruses to a sufficient read depth and coverage (31). Taken together, these results highlight the current clinical importance of metagenomic next-generation sequencing as that is increasingly regarded as a useful and complementary technique for the detection of pathogens that could not be routinely detected by current diagnostic tests (32); this is in addition to the pathogen-targeted molecular assays that are especially crucial for the differentiation between genetically closely related microorganisms, such as enteroviruses, rhinoviruses, and parechoviruses (33–36). Whether viral co-infections are associated with increased disease severity compared to monoinfections is still controversial in the literature. Loevinsohn et al. (37) reported that infection with multiple viruses is not associated with severe disease when compared to monoinfections. Nevertheless, in RSV-infected patients, co-infections with other viruses were associated with diarrhea, as well as longer duration of influenza-like illness symptoms (37). On the other hand, another study has found that hospital admission odds are significantly increased in influenza-positive patients co-infected with respiratory viruses, except for co-infection with rhinovirus/enterovirus (38). According to Wu et al. (39), co-infection of influenza and rhinovirus/enterovirus might be protective in infected patients.

In this study, we have found that the microbiota of patients with clades 5a.2a had a higher mean of alpha diversity as well as that they have formed non-overlapping cluster with PCA. In the literature, no studies have elucidated the variation of nasopharyngeal microbiota between different clades of influenza viruses. The variation was only elucidated between infected patients and healthy controls.

Indeed, studies have shown that the microbial communities are significantly different between flu-infected and non-infected patients (40, 41). Moreover, in their study, Ding et al. (42) found a significant association between the infecting influenza type and the composition of the nasopharyngeal microbiota. They have shown that influenza A and B were associated with different microbial communities compared to healthy uninfected individuals.

In our study, the most common phyla detected were in accordance with a recent paper published in China (43). Similarly, the top identified genera were in accordance with previous studies that found that *Streptococcus*, *Neisseria,* and *Staphylococcus* are among the top genera observed in patients with influenza illness (43). Moreover, interestingly, our study has shown that *S. pneumoniae, S. aureus*, *H. influenzae,* and *M. catarrhalis* are among the top species detected in influenza samples. These opportunistic pathogens are the most common species known to cause bacterial superinfections in influenza patients in the literature. This is in addition to others such as *P. aeruginosa* and *Acinetobacter baumannii* (44). Bacterial secondary infections are regarded as one of the main causes of death and increased morbidity in influenza patients (45, 46). Indeed, Arranz-Herrero et al. (44) reported a 3.4-fold increase in mortality risk in patients who experienced bacterial superinfections compared to those who did not.

There were several limitations in this study. The first one is the incomplete metadata of old samples as well as those collected in 2022. As mentioned in Materials and Methods, samples collected in 2022 were from a referral laboratory, from different hospitals in Riyadh, which rendered the collection of metadata from different settings challenging. The incomplete metadata, in our study, hindered the full comparison of clinical characteristics with genome and metatranscriptomic results. Furthermore, the results regarding the comparison of the nasopharyngeal microbiota between different influenza A clade-infected patients and inpatients versus outpatients should be considered carefully. This is due to the small sample size and the uneven distribution of patients among different categories. Taken together, the study has shown that the epidemiology of influenza A is changing from 6B.1 to 5a.2a. A mutation S247N, mediating antiviral resistance, was identified and should be carefully monitored in other studies throughout KSA. The major influence on nasopharyngeal microbiota was the infecting clade of the influenza virus. Future studies should aim at exploring the nasopharyngeal microbiota composition between healthy subjects and infected patients with different clades of influenza and with a larger sample size. This is in order to give more insight into the dynamics of the microbiota upon infection with different types and/or clades of the influenza A virus.

## AFTER EPUB

*This article was published on 21 June 2024 with errors in the affiliation for Bader Alhatlani. The affiliation was corrected in the current version, posted on 9 July 2024.]*

## SUPPLEMENTAL MATERIAL

Supplemental material spectrum.00665-24-s0001.docx Tables S1 to S4; Fig. S1.

ASM does not own the copyrights to Supplemental Material that may be linked to, or accessed through, an article. The authors have granted ASM a non-exclusive, world-wide license to publish the Supplemental Material files. Please contact the corresponding author directly for reuse.
