# Supplementary material for: Molecular surveillance of influenza A virus in Saudi Arabia: whole-genome sequencing and metagenomic approaches
Source: Microbiol Spectr. 2024 Jun 21;12(8):e00665-24. doi: 10.1128/spectrum.00665-24 (PMC11302342; doi:10.1128/spectrum.00665-24)
Supplement: Supplemental material — Tables S1 to S4; Fig. S1. [file spectrum.00665-24-s0001.docx]

**Supplementary table 1.** Primers used in this study

| **Primer** | **Primer sequence (5’ -> 3')** | **Amplicon Size (bp)** |
| --- | --- | --- |
| H1N1-PB2-F | AGCAAAAGCAGGTCAATTATATTC | 2341 |
| H1N1-PB2-R | AGTAGAAACAAGGTCGTTTTTAAAC |  |
| H1N1-PB1-F | AGCAAAAGCAGGCAAACCATTTGAATG | 2341 |
| H1N1-PB1-R | AGTAGAAACAAGGCATTTTTTCATGAAGGAC |  |
| H1N1-PA-F | AGCAAAAGCAGGTACTGATCCGAAATG | 2333 |
| H1N1-PA-R | AGTAGAAACAAGGTACTTTTTTGGACAGTATGG |  |
| H1N1-HA-F | AGCAAAAGCAGGGGAAAATAAAA | 1778 |
| H1N1-HA-R | AGTAGAAACAAGGGTGTTTT |  |
| H1N1-NP-F | AGCAAAAGCAGGGTAGATAATCAC | 1565 |
| H1N1-NP-R | AGTAGAAACAAGGGTATTTTTCTTTAATTGTC |  |
| H1N1-NA-F | AGCGAAAGCAGGAGTTTAAAATG | 1565 |
| H1N1-NA-R | AGTAGAAACAAGGAGTTTTTTG |  |
| H1N1-M-F | AGCGAAAGCAGGTAGATATTGAAAGA | 1027 |
| H1N1-M-R | AGTAGAAACAAGGTAGTTTTTTACTC |  |
| H1N1-NS-F | AGCAAAAGCAGGGTGACAAAAAC | 890 |
| H1N1-NS-R | AGTAGAAACAAGGGTGTTTTTTAGTACTAAATAAG |  |

**Supplementary table 2.** Primers used in this study

| **Gene Segment** | **PCR Conditions** |
| --- | --- |
| PB1, PA | 94 ◦C, 4 min; 94 ◦C, 30 s; 63 ◦C, 30 s; 72 ◦C, 4 min for 35 cycles; 72 ◦C, 7 min |
| PB2, NS | 94 ◦C, 4 min; 94 ◦C, 30 s; 58 ◦C, 30 s; 72 ◦C, 4 min for 35 cycles; 72 ◦C, 7 min |
| HA | 94 ◦C, 4 min; 94 ◦C, 20 s; 48 ◦C, 30 s; 72 ◦C, 4 min for 35 cycles; 72 ◦C, 7 min |
| NA | 94 ◦C, 4 min; 94 ◦C, 20 s; 46 ◦C, 30 s; 72 ◦C, 4 min for 35 cycles; 72 ◦C, 7 min |
| NP, M | 94 ◦C, 4 min; 94 ◦C, 20 s; 43 ◦C, 30 s; 72 ◦C, 4 min for 35 cycles; 72 ◦C, 7 min |

**Supplementary table 3.** Demographic characteristics of the studied population.

| **Sample #** | **Gender** | **Age (Years)** | **Nationality** |
| --- | --- | --- | --- |
| **2** | M | 17 | Saudi |
| **5** | F | 40 | Indian |
| **6** |  |  |  |
| **7** | F | 93 | Saudi |
| **9** | F | 23 | Saudi |
| **10** | F | 51 | Saudi |
| **11** | M | 36 | Saudi |
| **12** | M | 29 | Saudi |
| **13** | F | 28 | Saudi |
| **26** | F | 58 | Saudi |
| **27** | M | 3 | Saudi |
| **31** | F | 41 | Saudi |
| **33** | F | 41 | Filipino |
| **34** | F | 26 | Saudi |
| **36** | F | 1 | Indian |
| **39** | F | 31 | Non-Saudi |
| **41** | F | 35 | Saudi |
| **49** | M | 28 | Saudi |
| **53** | F | 44 | Saudi |
| **58** | F | 27 | Saudi |
| **64** | M | 2 | Saudi |
| **76** | M | 50 | Saudi |
| **78** | F | 32 | Saudi |
| **81** | M | 70 | Saudi |
| **83** | F | 3 | Saudi |
| **89** | M | 60 | Saudi |
| **95** | F | 28 | Non-Saudi |
| **98** | M | 1 | Saudi |
| **99** | F | 58 | Saudi |
| **102** | M | 25 | Saudi |
| **103** | F | 30 | Saudi |
| **74** | M | 14 | Saudi |
| **N1** | M | 48 | Saudi |
| **N2** | F | 67 | Saudi |
| **N8** | F | 10 | Saudi |
| **N9** | M | 36 | Saudi |
| **N10** | M | 5 | Saudi |
| **N11** | M | 15 | Saudi |
| **N13** | F | 64 | Saudi |
| **N14** | F | 67 | Saudi |
| **N15** |  | 7 | Non-Saudi |
| **N17** | M | 35 | Saudi |
| **N18** | M | 56 | Saudi |
| **N19** | F | 3 | Yemeni |

F = female, M = male, # = number, N = new samples collected in 2022, Blanks = data not available

**Table 4.** Resistance and virulence genes detected in this study.

| **Sequence** | **Gene** | **Coverage  Percent** | **Percent  Identity** | **Database** | **Accession** | **Product** |
| --- | --- | --- | --- | --- | --- | --- |
| 8be32c63-fa92-4e79-8bcf-f602c0db7e70 | RLMH | 83.54 | 81.35 | Megares | MEG_6058 | Drugs:MLS:23S_rRNA_methyltransferases:RLMH |
| 16697b46-08ef-462a-b923-893a3310a052 | cap8E | 95.72 | 95.26 | VFDB | NP_644943 | Capsular polysaccharide synthesis enzyme Cap8E [Capsule (VF0003)] [Staphylococcus aureus subsp. aureus MW2] |
| a631ee9b-0cec-4b85-96d1-d75f89a4a95a | clpP | 93.47 | 75.31 | VFDB | NP_465991 | ATP-dependent Clp protease proteolytic subunit [ClpP (VF0074)] [Listeria monocytogenes EGD-e] |


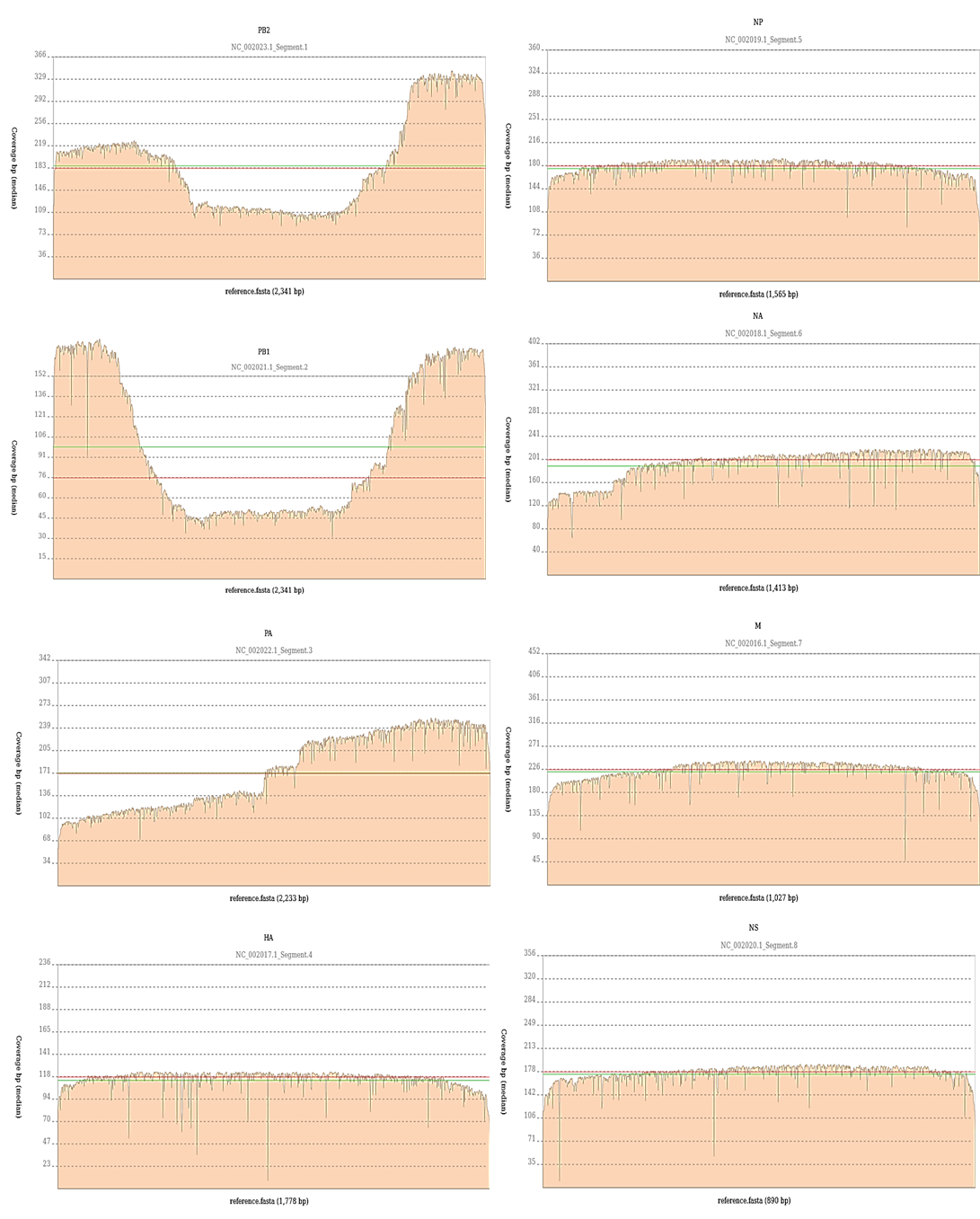


**Supplementary figure 1.** Coverage of aligned reads across each influenza A segment for N15 sample. The Y axis represents the number of reads covering the reference sequence for each influenza A segment and the X axis shows the position in the corresponding segment. Green line represents the mean, and red line the median depth.
